# Sulphur-containing amino acids promote the expression of *CG33474* and its neighbouring genes through the transsulphuration pathway

**DOI:** 10.1080/19336934.2026.2650576

**Published:** 2026-04-12

**Authors:** Lulin Xu, Meng Liu, Li He

**Affiliations:** a The First Affiliated Hospital of USTC, Division of Life Sciences and Medicine, University of Science and Technology of China, Hefei, China; b Anhui Key Laboratory of Tissue Transplantation, Department of Histology and Embryology, School of Basic Medicine, Bengbu Medical University, Bengbu, China

**Keywords:** Sulphur-containing amino acids, transsulphuration pathway, *CG33474*, antioxidant effect, cis-regulatory element

## Abstract

Sulphur-containing amino acids (SAAs), including methionine and cysteine, play crucial roles in antioxidant defence, anti-ageing, cytoprotection, and anti-inflammatory responses. Previous studies have shown that SAAs promote peroxisome elevation and fat loss by inducing the expression of the peroxisome-related gene *CG33474* in the *Drosophila* fat body. However, the underlying regulatory mechanism remains unclear. In this study, we demonstrated that the transsulphuration pathway contributes to *CG33474* induction, as supplementation with specific downstream metabolites of this pathway recapitulates this effect. Moreover, we found that SAAs upregulate not only *CG33474* but also several neighbouring genes – including *CG11825*, *Prx2540-1*, *Prx2540-2*, and *CG12896* – suggesting coordinated regulation within this genomic locus. Through fluorescence reporter assays, we discovered that a ~1 kb genomic region upstream of *CG33474* harbours the cis-regulatory element mediating SAA responsiveness and that this responsiveness is fat body-specific. Finally, our data suggest that induction of *CG33474* may play a role in resistance to different stresses and in regulating ageing as fat body-specific overexpression of *CG33474* significantly extends lifespan in *Drosophila*. Together, our findings reveal that SAAs modulate the expression of *CG33474* and its adjacent genes through the transsulphuration pathway, providing an additional mechanistic basis for the antioxidant effects of SAAs.

## 1. Introduction

Protein is one of three essential macronutrients for animals. Among 20 canonical amino acids, methionine and cysteine are classified as sulphur-containing amino acids (SAAs). SAAs play crucial roles in a wide range of physiological and pathological processes, including metabolism [1], immune regulation, tumourigenesis [2], and ageing [3]. Sulphur-containing amino acid residues in proteins can react with intracellular oxidants (such as H_2_O_2_ and O^2-^). These oxidative modifications can be reversed through cellular reduction systems that consume reducing molecules like NADPH, representing a key redox-sensitive post-translational modification (PTM) that modulates protein function [4].

Additionally, SAAs contribute to the generation of S-adenosylmethionine (SAM). SAM is not only the primary methyl donor for methylation reactions involving proteins, DNA, and RNA but also a synthetic precursor for several metabolites, such as polyamines and glutathione (GSH) [5]. Methionine also acts as a metabolic precursor for cysteine via the transsulphuration pathway. This conversion ultimately yields a variety of downstream metabolites, including α-ketobutyrate, taurine, GSH, sulphate, and hydrogen sulphide (H_2_S) [6]. Several of these compounds possess antioxidant properties, enabling them to scavenge reactive oxygen species (ROS) and protect cells from oxidative damage. Notably, tumour cells are particularly dependent on cysteine metabolism due to their higher ROS levels [7]. Among these metabolites, H_2_S has emerged as an important gaseous signalling molecule with diverse biological activities, including antioxidant, anti-inflammatory, vasorelaxant, and neuroprotective effects [8]. H_2_S has been proposed to enhance GSH regeneration, inhibit ROS production, activate K^+^ channels, modulate mitochondrial electron transport to maximise ATP generation, and regulate neurotransmitter/hormone secretion [8–12]. Nevertheless, the precise molecular mechanisms underlying many of these H_2_S-mediated effects remain incompletely understood.

Previous studies have shown that dietary SAAs promote peroxisome elevation and fat loss by inducing *CG33474* expression in the *Drosophila* fat body [13], with the transcriptional regulatory mechanism as well as biological functions remaining elusive. In this study, we showed that SAAs induce *CG33474* expression likely through downstream metabolites of the transsulphuration pathway. We also noticed that dietary SAAs not only upregulate the peroxisome-related gene *CG33474* but also induce the expression of multiple genes, including *CG12896, Prx2540-1, Prx2540-2*, and *CG11825*, all of which are located around the *CG33474* locus, suggesting potential co-regulation possibly mediated by shared cis-regulatory elements. Using reporter assays, we mapped this cysteine-responsive cis-regulatory region to a ~1 kb segment upstream of *CG33474*. Finally, we provided evidence that *CG33474* expression is associated with the antioxidant effects conferred by dietary SAAs.

## 2. Materials and methods

### 2.1. Drosophila culture

Flies were kept on a standard cornmeal food (per litre: 88 g glucose, 56 g cornmeal, 40 g yeast, 5.5 g agar, and 1 litre water, supplemented with 12.5 ml 10% sodium methylparaben solution and 10 ml propionic acid as preservatives) at 25°C and 60% humidity under a 12-hour light/12-hour dark cycle.

Drug treatments utilised in this study: 25 mM key compounds from the transsulphuration pathway; 25 mM NaHS (as an H_2_S donor); 25 mM individual amino acids; 25 mM N-acetyl-L-cysteine (NAC, Beyotime); diallyl trisulphide (DATS, Yuanye) at 2, 4, 5, or 10 mM; allicin (Macklin), diallyl disulphide (DADS, Macklin), and dimethyl trisulphide (DMTS, Macklin) at 5, 10, or 20 mM; 20 mM paraquat (Sigma); 5% H_2_O_2_ (Sinopharm). A piece of 11 cm double-ring qualitative filter paper (Cytiva) was cut into four equal sectors through the centre and one sector was placed into an empty fly culture tube (Biosharp). For liquid drug-containing medium preparation, the drug was dissolved in ddH_2_O or 5% sucrose (Sigma) to prepare specific concentrations. 750 μl of the drug solution was pipetted onto the filter paper to ensure complete soaking. For solid drug-containing medium preparation, the drug was dissolved in 1% agarose (BioFroxx), rapidly dispensed into fly tubes (5 ml per tube), and allowed to solidify at room temperature.

Notably, although 25 mM methionine and 25 mM cysteine exceed the physiological levels typically found in standard *Drosophila* diets, these concentrations were selected to maximize the biological response for mechanistic studies. Adult flies at 5–7 days post-eclosion or early third-instar larvae were used in this study.

The following fly strains were used: *w*^*1118*^ (lab stock), *Canton S* (lab stock), *CG33474-Gal4* (lab stock), *CG33474-GFP* (this study), *CG33474-Gal4,UAS-GFP* (this study), *CG33474-Gal4,UAS-RFP* (this study), *P1-mRuby* (this study), *P2-mRuby* (this study), *P3-mRuby* (this study), *P4-mRuby* (this study), *P5-mRuby* (this study), *P6-mRuby* (this study), *ppl-Gal4* (BDSC, #58768), *UAS-CG33474* (lab stock), *UAS-CG33474-RNAi* (BDSC, #64672), *lpp-Gal4* (BDSC, #94567), *UAS-Luciferase-RNAi* (BDSC, #31603), *UAS-CBS-RNAi* (BDSC, #41877), *UAS-CSE-RNAi* (BDSC, #41876), *UAS-TST1-RNAi* (BDSC, #62215).

### 2.2. Fluorescence imaging of Drosophila

Adult flies were either directly placed on a CO_2_-anaesthesia pad (Kaisi) or transferred to 1.5 ml microcentrifuge tubes (Biosharp) for 30 min at −20°C. Larvae were washed with PBS and placed in 1.5 ml tubes containing 500 μl absolute ethanol (Sinopharm) for 30 min at −20°C. Then these dormant flies or larvae in tubes were transferred onto glass slides (Sail Brand) and observed under the Leica M205 FCA microscope.

### 2.3. RT-qPCR

Flies were collected into 1.5 ml RNase-free micro tubes (Axygen) containing 500 μl TRIzol Reagent (Biosharp) and lysed using grinding rods (Sangon Biotech). After centrifuging at 12,000 × *g* for 5 min at 4°C, the supernatant was transferred to a new 1.5 ml RNase-free tube, mixed with 100 μl chloroform (Sinopharm), vortexed thoroughly, and incubated on ice for 5 min. After a centrifugation at 12,000 × *g* for 15 min at 4°C, 180 μl of the aqueous phase was carefully transferred to a new 1.5 ml RNase-free tube and mixed with an equal volume of ice-cold isopropanol (BBI). The mixture was incubated on ice for 10 min, and centrifuged at 12,000 × *g* for 10 min at 4°C. The RNA pellet was washed twice with 500 μl ice-cold 75% ethanol, each followed by a centrifugation at 7,500 × *g* for 5 min at 4°C. After drying to a translucent state, the RNA was dissolved in DEPC water (Biosharp). RNA concentrations were measured with a NanoDrop 2000 spectrophotometer, and then diluted to 100 ng/μl. cDNA synthesis was performed using the Hifair III 1st Strand cDNA Synthesis SuperMix for qPCR kit (gRNA digester plus) (Yeasen). 1 μg total RNA (10 μl) was mixed with 3 μl 5 × gDNA digester Mix and 2 μl RNase-free water for 2 min at 42°C. After removing genomic DNA, 5 μl 4 × Hifair III SuperMix Plus was added, and the reaction was incubated at 25°C for 5 min, 55°C for 15 min, and 85°C for 5 min to obtain the cDNA for qPCR. RT-qPCR was performed using the PerfectStart Green qPCR SuperMix kit (Transgen) on a QuantStudio 3 System (Thermo Scientific) with the cycling parameters: initial denaturation at 94°C for 30 sec; 40 cycles of 94°C for 5 sec and 60°C for 30 sec. The relative mRNA levels were calculated with the 2^−ΔΔCt^ method and normalised to *RpL23* (*Drosophila*). Three independent biological replicates were performed and the primer sequences used in RT-qPCR are listed in Supplementary Table S1.

### 2.4. Genomic DNA extraction

*Drosophila* genomic DNA was extracted using the FastPure Blood/Cell/Tissue/Bacteria DNA Isolation Mini Kit (Vazyme). Five adult flies were collected into a 1.5 ml microcentrifuge tube and ground with a grinding rod. The sample was sequentially mixed with 200 μl Buffer ACL and 20 μl Proteinase K by vortexing, followed by incubation at 56°C for 30 min. After adding 200 μl Buffer BCL and vortexing, 150 μl anhydrous ethanol was added and vortexed thoroughly. The mixture was transferred to a column placed in a 2 ml collection tube. After a 15-min incubation at RT, the column was centrifuged at 13,400 ×* g* for 1 min. The filtrate was discarded, and the column was placed back into the collection tube. Then, 500 μl Buffer WA was added to the column, followed by a centrifugation at 13,400 × *g* for 1 min. The filtrate was discarded, and the column was placed back into the collection tube. 600 μl Buffer WB was added into the column, followed by a centrifugation at 13,400 ×* g* for 1 min. This Buffer WB wash step was repeated twice. The filtrate was discarded again, and the column was then centrifuged at 13,400 × *g* for 2 min. After leaving the column open at room temperature for 2–5 min to remove residual ethanol, the column was transferred to a new 1.5 ml microcentrifuge tube, and 50 μl ddH_2_O was added to the column. After a 10-min incubation at RT, DNA was eluted by a centrifugation at 13,400 ×* g* for 2 min.

### 2.5. Construction of fluorescence reporters

The pCaSpeR4 vector (lab stock) was linearised with QuickCut™ BamHI (Takara) and the product was purified using the SanPrep Column PCR Product Purification Kit (Sangon Biotech). The DNA sequence from approximately 4.2 kb upstream to approximately 2.6 kb downstream of *CG33474* was divided into six segments, each approximately 1 kb. These segments were PCR-amplified from *w*^*1118*^ genomic DNA. Each segment was recombined with the linearised vector, followed by transformation, colony PCR, plasmid extraction, and DNA sequencing. The resulting fluorescent reporter plasmids were named as P1, P2, P3, P4, P5, and P6. These plasmids were then injected into the embryos of the *y*^*1*^ *w*^*67c23*^;* P{CaryP}attP40 Drosophila* strain (injection performed by UniHuaii) to generate transgenic flies. The injected *Drosophila* embryos were designated as F0. After eclosion of the F0 flies, virgin females with yellow eyes were crossed with yellow-eyed males. Their offspring were designated as F1. After eclosion of the F1 flies, virgin females with orange eyes were crossed with orange-eyed males to establish stable transgenic *Drosophila* lines.

### 2.6. Immunostainings

For immunostaining of *Drosophila* tissues, flies were dissected in PBS. Dissected tissues were transferred to 1.5 ml microcentrifuge tubes containing 500 μl 4% paraformaldehyde (PFA, Sangon Biotech) and fixed for 30 min at room temperature (RT). After three 10-min washes with 500 μl PBST (0.05% Triton X-100 in PBS) at RT, samples were blocked with 500 μl 2% bovine serum albumin (BSA, Sangon Biotech) in PBST (PBSTB) for 30 min at RT. Samples were then incubated with the primary antibody diluted in PBSTB at 4°C overnight or at RT for 2 h. After three 10-min washes with 500 μl PBST, samples were incubated with the Alexa Fluor-conjugated secondary antibody and DAPI (1:1000 dilution in PBSTB) for 2 h at RT followed by the same washing procedures above. Samples were then mounted with antifade mounting agent (4% N-Propyl Gallate, 90% Glycerol, PBS) for imaging (Leica DMi8).

### 2.7. Stress resistance and lifespan assays

For the stress resistance assay, newly eclosed flies of the designated genotypes were collected into fly tubes for mating. After 5 days, female flies with similar body size were selected and subjected to paraquat, hydrogen peroxide, and starvation. Four tubes were used per treatment group and 20 female flies were placed in each tube. The number of dead flies was recorded at 9:00, 12:00, 16:00, 20:00, and 23:00 daily for survival curve generation. For the lifespan assay, newly eclosed flies of the designated genotypes were collected into fly tubes for mating. After 5 days, female flies with similar body size were selected. Four tubes were used per treatment group and 20 female flies were placed in each tube containing a standard cornmeal food. Every 5 days, flies were transferred to new fly tubes, and the number of dead flies was recorded at 23:00 daily for survival curve generation.

### 2.8. RNA-Seq

*w*^*1118*^ female flies under different treatments were collected into 1.5 ml RNase-free micro tubes containing 500 μl TRIzol Reagent and lysed using grinding rods. Subsequent steps were conducted by GENEWIZ Corporation, including RNA extraction, quality assessment of RNA samples, library construction, library purification, library detection, library quantification, and sequencing. The main bioinformatic analyses were performed as follows. Raw sequencing reads were first subjected to quality control: technical sequences (adapters, PCR primers, or fragments thereof) and bases with quality scores lower than 20 were removed using Cutadapt (v1.9.1). The high-quality clean reads were then aligned to the *Drosophila melanogaster* reference genome sequence (GCF_000001215.4) using Hisat2 (v2.2.1). Gene and isoform expression levels were quantified using HTSeq (v0.6.1) and differential gene expression analysis was performed using the DESeq2 package with an adjusted *p*-value (padj) ≤ 0.05. Functional enrichment analyses of the differentially expressed genes were conducted for Gene Ontology (GO) terms and Kyoto Encyclopedia of Genes and Genomes (KEGG) pathways.

### 2.9. Statistical analysis

Statistical analysis was performed by GraphPad Prism 10, and the results are expressed as mean ± standard deviation (SD). Statistical significance was assessed using two-tailed Student’s t-test, Fisher’s exact test, one-way ANOVA, two-way ANOVA, or log rank test and was indicated with * *p* < 0.05, ** *p* < 0.01, *** *p* < 0.001, **** *p* < 0.0001, ns, not significant.

## 3. Results

### 3.1. Downstream metabolites of the transsulphuration pathway mediating cysteine- and methionine-induced expression of CG33474

**Figure 1.**
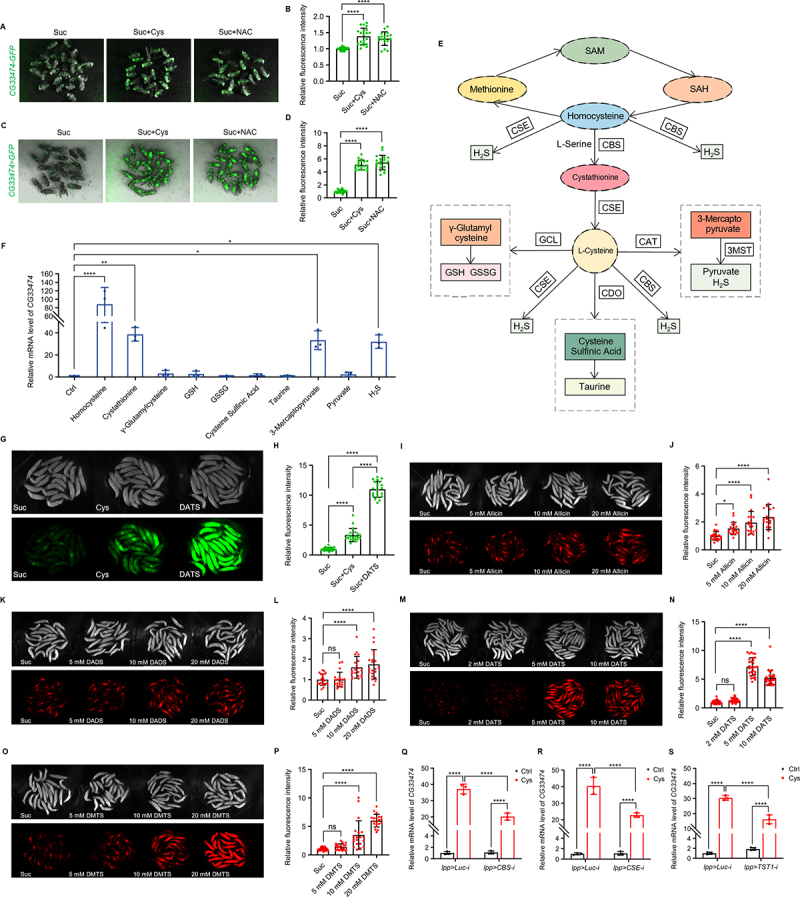
Downstream metabolites of the transsulphuration pathway mediating cysteine- and methionine-induced expression of *CG33474*. (A-B) representative images (A) and quantification (B, from left to right: 23, 21, and 18 flies) of *CG33474-GFP* female flies expressing GFP 36 h following the designated treatments. Suc: 5% sucrose; Suc + Cys: 5% sucrose + 25 mM cysteine; Suc + NAC: 5% sucrose + 25 mM N-acetyl-L-cysteine. (C-D) representative images (C) and quantification (D, from left to right: 20, 20, and 20 flies) of *CG33474-Gal4,UAS-GFP* female flies expressing GFP 36 h following the designated treatments. Suc: 5% sucrose; Suc + Cys: 5% sucrose + 25 mM cysteine; Suc + NAC: 5% sucrose + 25 mM N-acetyl-L-cysteine. (E) a diagram represents the transsulphuration pathway. (F) relative *CG33474* mRNA levels in *w^1118^* female flies 36 h fed with Ctrl (ddH_2_O) or key compounds from the transsulphuration pathway (25 mM). *n* = 3. (G-H) representative images (G) and quantification (H, from left to right: 20, 20, and 20 larvae) of *CG33474-Gal4,UAS-GFP* larvae expressing GFP 36 h following the designated treatments. Suc: 5% sucrose; Suc + Cys: 5% sucrose + 25 mM cysteine; Suc + DATS: 5% sucrose + 4 mM diallyl trisulphide. (I-P) representative images (I, K, M, O) and quantification (J, L, N, P) of *CG33474-Gal4,UAS-RFP* larvae expressing RFP 36 h following the designated treatments. Suc: 5% sucrose; various concentrations of allicin (Suc + allicin), DADS (Suc + DADS), DATS (Suc + DATS), or DMTS (Suc + DMTS). (J) from left to right: 21, 20, 20, and 20 larvae. (L) from left to right: 20, 20, 20, and 21 larvae. (N) from left to right: 30, 30, 30, and 30 larvae. (P) from left to right: 21, 20, 20, and 20 larvae. (Q-S) relative *CG33474* mRNA levels in *lpp-Gal4 > UAS-Luciferase-RNAi* (control), *lpp-Gal4 > UAS-CBS-RNAi*, *lpp-Gal4 > UAS-CSE-RNAi*, or *lpp-Gal4 > UAS-TST1-RNAi* larvae 36 h following the designated treatments. Ctrl: 5% sucrose; Cys: 5% sucrose + 25 mM cysteine. *n* = 3. One-way ANOVA (B, D, F, H, J, L, N, P) and two-way ANOVA (Q, R, S) were performed. * *p* < 0.05; ** *p* < 0.01; **** *p* < 0.0001; ns, not significant.

Our previous study has shown that SAAs (methionine and cysteine) specifically induce the expression of *CG33474* in *Drosophila melanogaster* [13]. To better visualise *CG33474* expression *in vivo*, we generated a *CG33474-GFP* knock-in line. Female flies carrying this allele were fed with sucrose, cysteine, or N-acetyl-L-cysteine (NAC), a cell-permeable cysteine precursor [14], for 36 h. Both cysteine and NAC significantly increased GFP fluorescence (Figure 1(A,B)). However, fluorescence driven by the endogenous promoter remained relatively weak. To amplify the signal, we constructed a *CG33474-Gal4,UAS-GFP* fluorescent reporter line, in which GFP expression is amplified via the Gal4/UAS system [15]. *CG33474-Gal4,UAS-GFP* female flies were fed with sucrose, cysteine, or NAC for 36 h, and robust GFP fluorescence was detected specifically under cysteine or NAC conditions (Figure 1(C,D)). These results suggest that cysteine induces *CG33474* expression and that NAC induces *CG33474* expression through its metabolism to cysteine.

Methionine can be metabolised to cysteine via the transsulphuration pathway, generating multiple downstream metabolites. We hypothesised that SAAs may regulate *CG33474* expression through shared downstream metabolites. To test this, we fed *w*^*1118*^ female flies with key metabolites of the transsulphuration pathway for 36 h and quantified *CG33474* mRNA levels using RT-qPCR. Both H_2_S (NaHS was dissolved in ddH_2_O to generate H_2_S) and H_2_S-generating metabolites of the transsulphuration pathway – including homocysteine, cystathionine, and 3-mercaptopyruvate – significantly upregulated *CG33474* mRNA levels. In contrast, metabolites associated with alternative branches of cysteine metabolism – including GSH, taurine, and pyruvate – failed to induce *CG33474* expression (Figure 1(E,F)). These data suggest that the transsulphuration pathway may regulate *CG33474* expression via its specific downstream metabolites.

To investigate the role of H_2_S as a downstream effector of the transsulphuration pathway in vivo, we fed *CG33474-Gal4,UAS-GFP* larvae with sucrose, cysteine, or diallyl trisulphide (DATS) – a natural H_2_S donor that rapidly releases H_2_S [16] – for 36 h. Both cysteine and DATS markedly increased GFP fluorescence, with DATS producing a stronger induction than cysteine (Figure 1(G,H)). Furthermore, we fed *CG33474-Gal4,UAS-RFP* larvae with sucrose and different natural H_2_S donors – including allicin, diallyl disulphide (DADS), DATS, and dimethyl trisulphide (DMTS) – for 36 h. Among these, allicin and DMTS are garlic-derived compounds, whereas DADS and DATS are allicin derivatives. Allicin (5, 10, and 20 mM), DADS (10 and 20 mM), and DMTS (10 and 20 mM) significantly increased RFP fluorescence intensity (Figure 1(I–P)), while DATS at 5 and 10 mM also produced a significant induction (Figure 1(M,N)). Moreover, DATS exhibited a stronger *CG33474*-inducing ability than allicin and DADS, consistent with its superior H_2_S-releasing capacity due to a higher number of sulphur atoms. Additionally, DMTS showed a stronger induction than allicin and DADS, possibly due to its structural similarity to DATS, as both contain three sulphur atoms.

Additionally, we performed fat body-specific knockdown of *CBS*, *CSE*, or *TST1* in *Drosophila*. CBS and CSE are responsible for H_2_S synthesis via the transsulphuration pathway, while TST1, like 3-MST, belongs to the thiosulphate sulphurtransferase (TST) family and contributes to H_2_S biosynthesis. TST1 may transfer sulphur from a donor (e.g. thiosulphate) to form a persulphide intermediate, which can be reduced to H_2_S by thioredoxin, similar to the mechanism of 3-MST [17]. We fed *lpp-Gal4 > UAS-Luciferase-RNAi* (control), *lpp-Gal4 > UAS-CBS-RNAi*, *lpp-Gal4 > UAS-CSE-RNAi*, or *lpp-Gal4 > UAS-TST1-RNAi* larvae either a control diet or a cysteine-supplemented diet. After 36 h, we dissected the fat body and measured *CG33474* mRNA levels by RT-qPCR. Larvae of all four genotypes exhibited significant upregulation of *CG33474* expression by cysteine. However, compared with the control larvae, the cysteine-induced expression of *CG33474* was significantly attenuated in larvae with fat body-specific knockdown of *CBS*, *CSE*, or *TST1* (Figure 1(Q–S)). Taken together, our findings suggest that downstream metabolites of the transsulphuration pathway regulate cysteine- and methionine-induced expression of *CG33474*.

### 3.2. Cysteine and methionine induce the expression of multiple genes adjacent to CG33474 locus

**Figure 2.**
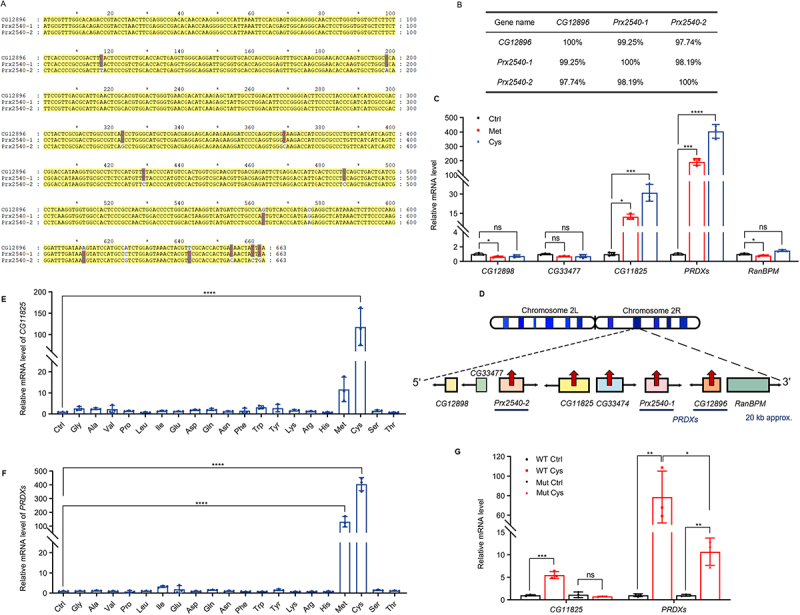
Cysteine and methionine induce the expression of multiple genes adjacent to *CG33474* locus. (A-B) coding sequence (CDS) base alignment results (A) and gene similarity analyses (B) of *CG12896*, *Prx2540-1*, and *Prx2540-2*. (C) relative mRNA levels of genes adjacent to *CG33474* in *w^1118^* female flies 36 h following the designated treatments. Ctrl: 1% agarose; Met: 1% agarose + 25 mM methionine; Cys: 1% agarose + 25 mM cysteine. *n* = 3. (D) a diagram represents the genomic locations of multiple genes adjacent to *CG33474*. (E-F) relative *CG11825* (E) and *PRDXs* (F) mRNA levels in *w^1118^* female flies fed with Ctrl (1% agarose) or various amino acids (1% agarose + 25 mM individual amino acids) for 36 h. *n* = 3. (G) relative *CG11825* and *PRDXs* mRNA levels in larvae 36 h following the designated treatments. Ctrl: 1% agarose; Cys: 1% agarose + 25 mM cysteine. *w^1118^* and *CG33474* homozygous mutants were used as WT (wild-type) and Mut (mutant), respectively. *n* = 3. One-way ANOVA (C, E, F) and two-tailed Student’s t-test (G) were performed. * *p* < 0.05; ** *p* < 0.01; *** *p* < 0.001; **** *p* < 0.0001; ns, not significant.

We then investigated how *CG33474* is transcriptionally upregulated. We hypothesised that a cysteine- and methionine-responsive cis-regulatory element exists in the genomic region near *CG33474*. To identify the cis-regulatory element, we first examined whether neighbouring genes are also responsive to cysteine or methionine. Due to the high similarity among *CG12896*, *Prx2540-1* and *Prx2540-2*, with only a few bases of the difference, and their annotation in FlyBase as homologs of human peroxiredoxin-6 (*PRDX6*), we collectively referred to these three genes as *PRDXs* (Figure 2(A,B)). To simultaneously quantify their expression, we designed a pair of qPCR primers targeting a conserved region shared by all three transcripts. We then fed *w*^*1118*^ female flies with control, methionine, or cysteine for 36 h and quantified the mRNA levels of genes adjacent to *CG33474* via RT-qPCR. Both *CG11825* and *PRDXs* showed significant upregulation under cysteine and methionine conditions (Figure 2(C)). Genomic mapping revealed that *CG11825* and the *PRDXs* cluster are located in close proximity to *CG33474* on chromosome 2R (Figure 2(D)). In contrast, more distal genes – including *CG12898*, *CG33477*, and *RanBPM* – were not significantly upregulated under either dietary condition (Figure 2(C)).

To determine whether *CG11825* and *PRDXs* exhibit the same amino acid specificity as *CG33474*, we fed *w*^*1118*^ female flies each of the 20 standard amino acids individually for 36 h and quantified *CG11825* and *PRDXs* mRNA levels by RT-qPCR. *CG11825* expression was significantly induced only by cysteine (Figure 2(E)), whereas *PRDXs* were robustly upregulated by both cysteine and methionine (Figure 2(F)). Previous studies have shown that cysteine induces *CG33474* expression more potently than methionine [13], which may account for the weaker response of *CG11825* to methionine.

Given that *CG11825* and *PRDXs* – like *CG33474* – respond specifically to cysteine, we hypothesised that *CG33474* may be functionally linked to these neighbouring genes. To test this, we used the *CG33474-Gal4* line as a *CG33474* homozygous mutant line because the Gal4 coding sequence is inserted into the *CG33474* coding region, disrupting endogenous *CG33474* expression. In previous studies, the mutation effect of this line has been verified through RT-qPCR [13]. We fed *w*^*1118*^ wild-type and *CG33474* homozygous mutant larvae either a control diet or a cysteine-supplemented diet for 36 h and measured *CG11825* and *PRDXs* mRNA levels by RT-qPCR. Cysteine-induced expression of both *CG11825* and *PRDXs* was significantly attenuated in *CG33474* homozygous mutants compared with wild-type controls (Figure 2(G)), suggesting that loss of *CG33474* impairs the cysteine-responsive activation of these genes.

### 3.3. Identification of the cis-regulatory element responsive to a cysteine-rich diet

**Figure 3.**
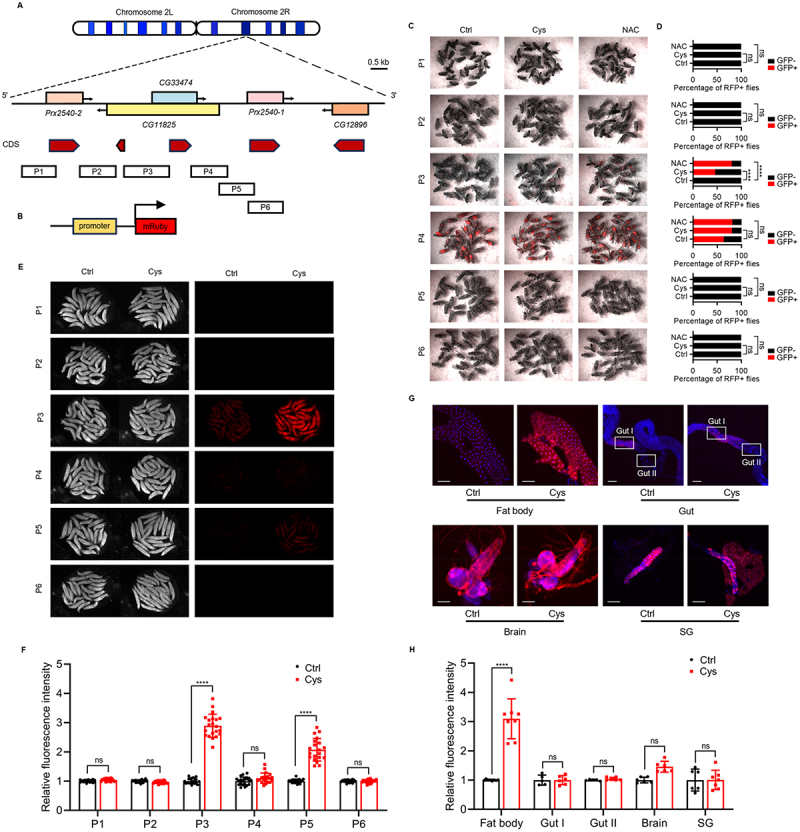
Identification of the cis-regulatory element responsive to a cysteine-rich diet. (A-B) (A) the DNA sequence from approximately 4.2 kb upstream to approximately 2.6 kb downstream of *CG33474* was divided into six segments: P1, P2, P3, P4, P5, and P6. (B) these segments were individually inserted into a linearised vector to construct the mRuby fluorescent reporter system. (C-D) representative images (C) and quantification (D) of P1, P2, P3, P4, P5 and P6 fluorescent reporter female flies expressing RFP 36 h following the designated treatments. Suc: 5% sucrose; Cys: 5% sucrose + 25 mM cysteine; NAC: 5% sucrose + 25 mM N-acetyl-L-cysteine. The number of flies counted in each group from left to right: P1: 27, 26, 24; P2: 28, 28, 23; P3: 29, 24, 25; P4: 27, 25, 31; P5: 28, 28, 25; P6: 28, 28, 28. (E-F) representative images (E) and quantification (F, from left to right: 20, 20; 20, 20; 20, 21; 20, 20; 20, 20; 20, 19 larvae) of P1, P2, P3, P4, P5 and P6 fluorescent reporter larvae expressing RFP 36 h following the designated treatments. Suc: 5% sucrose; Cys: 5% sucrose + 25 mM cysteine. (G) fluorescence microscopy images in the fat body, intestine, brain, and salivary glands of P3 fluorescent reporter larvae 36 h following the designated treatments. Suc: 5% sucrose; Suc + Cys: 5% sucrose + 25 mM cysteine. DAPI (blue) labelled nuclei, RFP fluorescence signals (red) indicate P3-driven expression. Scale bar: 100 µm. (H) quantification of relative RFP fluorescence intensity. From left to right: 8, 8; 5, 6; 5, 6; 6, 6; 6, 7 larvae. Fisher’s exact test (D) and two-way ANOVA (F, H) were performed. **** *p* < 0.0001; ns, not significant.

Cysteine and methionine induce the expression of *CG33474* and several adjacent genes, suggesting the presence of a cis-regulatory element responsive to SAAs. To identify this element, we divided the genomic region spanning approximately 4.2 kb upstream to 2.6 kb downstream of *CG33474* into six contiguous segments (P1-P6). Each segment was cloned into a linearised *mRuby* fluorescent reporter vector, generating six fluorescence reporter plasmids (Figure 3(A,B)). To better visualise *CG33474* expression in vivo, we injected these fluorescent reporter plasmids into *Drosophila* embryos and established homozygous transgenic lines. We then fed P1-P6 female flies with control, cysteine, or NAC for 36 h and observed RFP fluorescence expression. P3 female flies exhibited a significant increase in the proportion of RFP+ flies under cysteine or NAC conditions (Figure 3(C,D)). To further confirm these findings, we fed P1-P6 larvae with control or cysteine for 36 h. Consistently, dietary cysteine significantly enhanced the RFP fluorescence intensity in P3 larvae (Figure 3(E,F)). Although a modest increase in fluorescence was observed in P5 larvae under cysteine feeding, the effect was substantially weaker than that in P3 larvae. Crucially, the P5 line showed no significant change in the proportion of RFP+ adult females under cysteine or NAC conditions. In summary, through systematic dissection of the genomic region surrounding *CG33474*, we identified the P3 segment as harbouring the cysteine-responsive cis-regulatory element. This element likely mediates the transcriptional upregulation of *CG33474* and its neighbouring genes in response to SAAs.

To investigate whether the RFP fluorescence expression in P3 flies exhibits fat body specificity upon cysteine feeding, we fed P3 larvae either a control diet or a cysteine-supplemented diet. After 36 h, the larvae were dissected and RFP fluorescence signals in the fat body, gut, brain, and salivary glands were examined under the fluorescence microscope. Cysteine significantly enhanced RFP fluorescence intensity specifically in the fat body of P3 larvae, but failed to enhance it in the gut, brain, or salivary glands (Figure 3(G,H)). These findings indicate that the response of the cis-regulatory element within the P3 region to cysteine is fat body-specific.

### 3.4. CG33474 enhances stress resistance and extends lifespan in Drosophila

**Figure 4.**
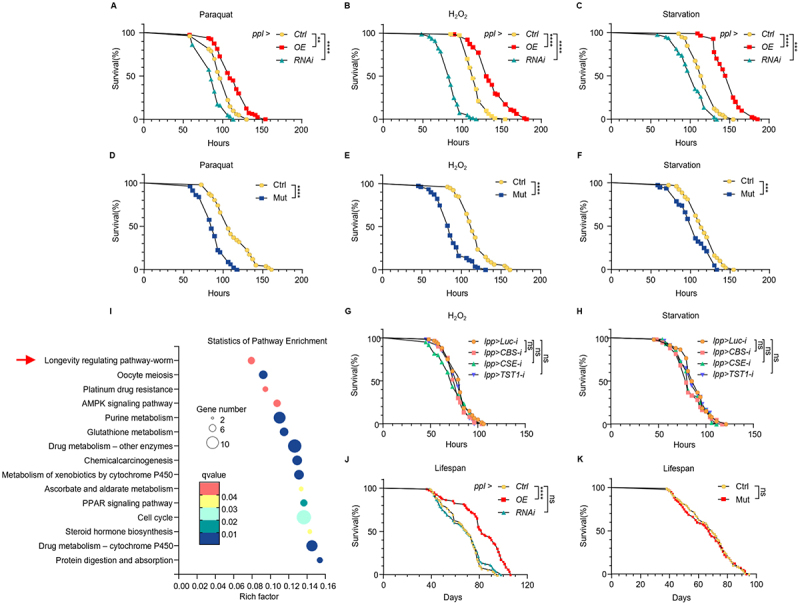
*CG33474* enhances stress resistance and extends lifespan in *Drosophila*. (A-C) survival of female flies with *CG33474* normal expression (Ctrl), overexpression (OE), or knockdown (RNAi) in the fat body under paraquat (A), H_2_O_2_ (B), or starvation (C) conditions. 80 flies per group. (D-F) survival of *w^1118^* (WT) and *CG33474* homozygous mutant (Mut) female flies under paraquat (D), H_2_O_2_ (E), or starvation (F) conditions. 80 flies per group. (G-H) survival of *lpp-Gal4 > UAS-Luciferase-RNAi* (control), *lpp-Gal4 > UAS-CBS-RNAi*, *lpp-Gal4 > UAS-CSE-RNAi*, or *lpp-Gal4 > UAS-TST1-RNAi* female flies under H_2_O_2_ (G) or starvation conditions (H). 60 flies per group. (I) KEGG pathway analysis of differentially expressed genes in *w^1118^* female flies 36 h following the designated treatments. Ctrl: 1% agarose; Met: 1% agarose + 25 mM methionine. The arrow indicates the longevity regulating pathway. *n* = 3. (J) survival of female flies with *CG33474* normal expression (Ctrl), overexpression (OE), or knockdown (RNAi) in the fat body. 80 flies per group. (K) survival of *w^1118^* (WT) and *CG33474* homozygous mutant (Mut) female flies. 80 flies per group. Log rank test was performed. ** *p* < 0.01; *** *p* < 0.001; **** *p* < 0.0001; ns, not significant.

Given that *CG33474* cooperates with *PRDXs* in response to SAAs and that *PRDXs* are homologous to human *PRDX6* which maintains cellular redox homoeostasis by reducing peroxide substrates [18,19], we hypothesised that *CG33474* may also play a role in antioxidant defence. To test this, we specifically overexpressed or knocked down *CG33474* in the fat body and subjected female flies to paraquat, hydrogen peroxide (H_2_O_2_), and starvation (paraquat and H_2_O_2_ are common inducers of oxidative stress, while starvation represents a metabolic stress condition). Fly mortality was recorded every 3–5 h. Fat body-specific knockdown of *CG33474* significantly reduced resistance to paraquat, H_2_O_2_, and starvation, whereas fat body-specific overexpression of *CG33474* markedly enhanced tolerance to these stressors (Figure 4(A–C)). Furthermore, we examined stress resistance in *w*^*1118*^ and *CG33474* homozygous mutant female flies under the same conditions. Compared to *w*^*1118*^ flies, *CG33474* homozygous mutant flies also showed significantly reduced survival under paraquat, H_2_O_2_, and starvation (Figure 4(D–F)). These findings indicate that elevated *CG33474* expression enhances stress resistance, whereas its reduction compromises the ability of *Drosophila* to cope with both oxidative and metabolic stress.

Considering that a downstream metabolite of the transsulphuration pathway, potentially H_2_S, plays an important role in SAA-induced *CG33474* expression, we also examined the effect of knocking down enzymes responsible for the production of H_2_S on stress resistance. We specifically knocked down CBS, CSE, or TST1 in the fat body and then subjected female flies of different genotypes to H_2_O_2_ and starvation. Compared with the control group, fat body-specific knockdown of these enzymes showed no significant effect on stress resistance (Figure 4(G,H)). This result is consistent with such knockdown failing to alter basal *CG33474* expression and only affecting SAA-induced *CG33474* expression.

Additionally, KEGG pathway analysis of RNA-seq data from *w*^*1118*^ female flies fed with control or methionine for 36 h demonstrated that dietary methionine significantly affects the longevity-regulating pathway (Figure 4(I)). To investigate whether *CG33474* influences lifespan, we monitored the survival of female flies with different genotypes under standard dietary conditions. Fat body-specific overexpression of *CG33474* significantly extended lifespan, whereas fat body-specific knockdown showed no significant effect (Figure 4(J)). Furthermore, compared with *w*^*1118*^ flies, *CG33474* homozygous mutant flies also showed no significant effect on lifespan (Figure 4(K)). Together, these results suggest that elevated expression of *CG33474* in the fat body promotes longevity, while its reduction or complete loss fails to significantly affect lifespan – consistent with the observation that *CG33474* is expressed at relatively low basal levels under normal conditions.

## 4. Discussion

Previous work in our laboratory has shown that SAAs (methionine and cysteine) promote peroxisome proliferation and fat loss by inducing the expression of *CG33474* in the *Drosophila* fat body. However, the molecular mechanism by which adipose cells sense SAAs and regulate specific gene expression remains to be elucidated. Using *Drosophila melanogaster* as a model, this study showed that SAAs induce the expression of *CG33474* and its neighbouring genes – *Prx2540-1*, *Prx2540-2*, *CG12896*, and *CG11825* – through specific downstream metabolites of the transsulphuration pathway, such as H_2_S, in a manner dependent on the cis-acting regulatory element located in the P3 region. Additionally, this study demonstrated that *CG33474* plays a role in resistance to different stresses and in regulating ageing.

Under standard dietary conditions, *w*^*1118*^ wild-type larvae exhibited Ct values of 25, 23.5, and 29 for *CG11825*, *PRDXs*, and *CG33474*, respectively, when the housekeeping gene Ct values were the same. *CG33474* displays a markedly lower basal expression level than *CG11825* and *PRDXs*, indicating that *CG33474* is expressed at low levels under normal physiological conditions. Therefore, knockdown or knockout of *CG33474* in flies under standard dietary conditions may show no overt phenotypes, whereas its overexpression may uncover latent physiological functions. For instance, the *CG33474* homozygous mutant line still develops and grows normally without obvious physiological defects, further indicating that *CG33474* is not essential for normal growth and development. This study found that fat body-specific overexpression of *CG33474* extends lifespan in *Drosophila*, whereas its knockdown or mutation shows no significant effect. All individual alleles used in this lifespan study were tested under corresponding experimental conditions. No significant lifespan changes were observed for these alleles prior to crossing (data not shown). However, a more precisely controlled experiment with a fat body specific GeneSwitch driver will be desirable in the future. Together with the finding that SAAs significantly induce *CG33474* expression, we hypothesise that *CG33474* may function as a nutritional signal-induced protective effector gene – characterised by low and non-essential expression under normal physiological conditions, but inducible by specific nutritional signals such as SAAs – thereby exerting roles in stress resistance and lifespan regulation.

This study has several limitations that warrant discussion. Although knockdown of CBS, CSE, or TST1 significantly attenuated SAA-induced *CG33474* expression, it failed to completely abolish the effect. We attribute this to the individual knockdown of CBS, CSE, or TST1: knocking down a single enzyme does not eliminate H_2_S production entirely. In the future, we plan to generate multi-gene knockout lines for H_2_S synthesis-related enzymes to further examine SAA-mediated induction of *CG33474*.

In addition, the molecular mechanisms by which H_2_S participates in signal transduction remain unclear. It is generally believed that one of the primary mechanisms through which H_2_S exerts its biological effects is protein persulphidation, an evolutionarily conserved oxidative post-translational modification [20]. Studies have reported that H_2_S influences the activity of target proteins by modifying protein cysteine residues [21]. Recent studies have shown that H_2_S-mediated persulphidation of HNF1α at Cys241 reduces its DNA-binding ability and subsequently decreases its transcriptional activity [22]. Moreover, H_2_S has been shown to activate the Nrf2 – a master regulator of antioxidant defence – by disrupting the Keap1-Nrf2 interaction through persulphidation of Keap1 at Cys151. Once activated, Nrf2 binds to antioxidant response elements (AREs) in the promoters of target genes, orchestrating the expression of multiple antioxidant enzymes [23]. Notably, during oxidative stress, PRDX1, PRDX5, and PRDX6 function through Nrf2-related signalling pathways to maintain cellular redox homoeostasis [24]. Given that *Drosophila PRDXs* are homologous to human *PRDX6* and cooperate with *CG33474* in response to SAAs, we speculate that Nrf2 or related transcription factors may mediate SAA-induced *CG33474* expression. Based on these findings, future studies are warranted to investigate whether H_2_S – a downstream metabolite of the transsulphuration pathway – modulates key transcription factors through persulphidation, thereby influencing their activity and ultimately regulating SAA-induced transcriptional activation of *CG33474*.

Moreover, while our data suggest a role for H_2_S in mediating SAA-induced *CG33474* expression, H_2_S levels were not directly measured in this study. A direct quantification of H_2_S production in vivo will be an important experiment for future investigations.

Finally, we identified the ~1 kb P3 region (Supplementary Figure S1) as containing the cis-regulatory element responsive to SAAs. We also analysed this P3 fragment for potential transcriptional factor binding sites using the MEME-Suite (Supplementary Table S2). However, this DNA sequence is relatively long and its internal structure and function warrant further investigation. Future studies aimed at finer mapping of the SAA-responsive element will help narrow down the critical genomic interval. Moreover, approaches such as yeast one-hybrid screening or DNA pull-down assays will be valuable for identifying the specific transcription factors that directly bind to this region and mediate SAA responsiveness.

## Supplementary Material

Supplemental Material

## Data Availability

All data generated or analysed in this study are included in the main text or the Supplementary Table S2. The raw data generated in this study are provided in the Source Data file in this paper.
