# Supplementary material for: Sulphur-containing amino acids promote the expression of CG33474 and its neighbouring genes through the transsulphuration pathway
Source: Fly (Austin). 2026 Apr 12;20(1):2650576. doi: 10.1080/19336934.2026.2650576 (PMC13078233; doi:10.1080/19336934.2026.2650576)
Supplement: Supplemental Material [file KFLY_A_2650576_SM7844.docx]

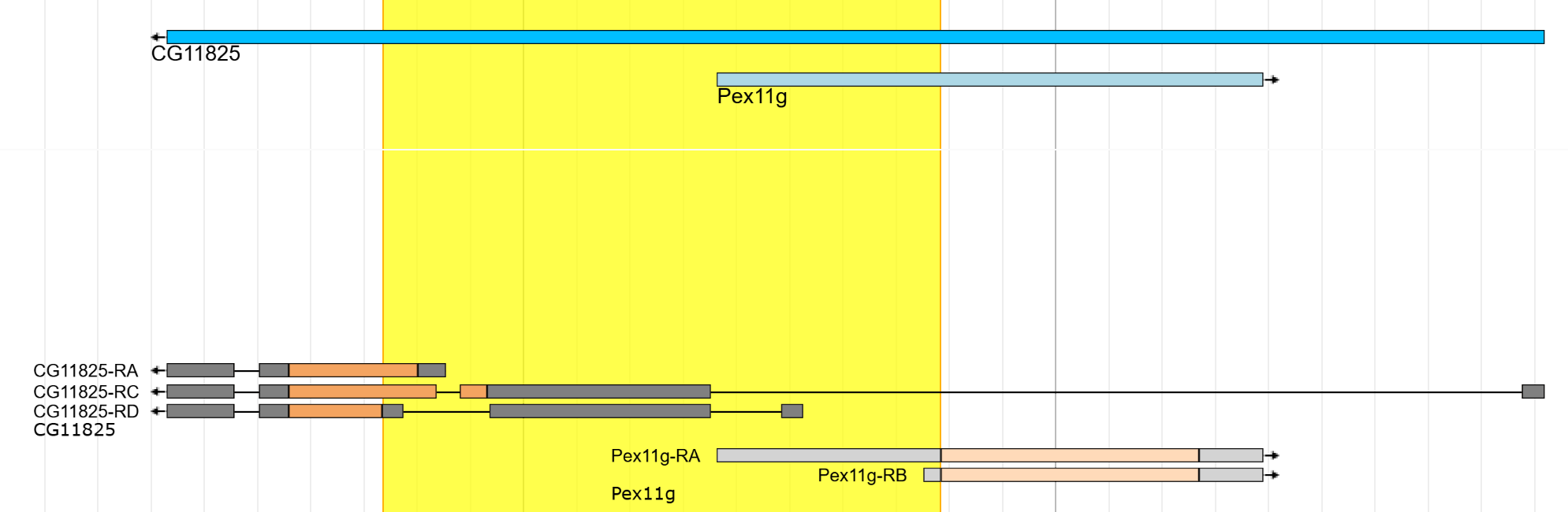


**Supplementary Fig. 1 Position of the P3 region in the *Drosophila* genome.**

The P3 region (colored in yellow) is located upstream of the *CG33474* coding sequence. The gene retains the official symbol *CG33474* in NCBI but has been updated to *Pex11g* (peroxisomal biogenesis factor 11 gamma) in FlyBase.

**Supplementary Table 1** Primer sequences utilized in RT-qPCR (*Drosophila*).

| Gene name | Forward primers (5'-3') | Reverse primers (5'-3') |
| --- | --- | --- |
| *CG33474* | TTGATTCCTGTAGAGCCCGAG | GGTCTGGACTCTGATTGATTTGG |
| *CG12898* | TTTAAGTGCCCCATCAAGCCA | CATCGACAACTGGAACCGTC |
| *CG33477* | TCGTGTCGTAAAGTTGGCTCC | CGCAACCCTTGAAGTTTTCGAT |
| *CG11825* | CTGGTGGGTATTGCCGGATT | TCCGGCTGTCAAACATCCAA |
| *PRDXs* | CCGTACCTAACTTCGAGGCC | CGAGTTCAATGCGTCAACGG |
| *RanBPM* | AAGTGCTTGTCCATCGGTTTG | GGGATCGGATAGGCAGTGC |
| *RpL23* | GACAACACCGGAGCCAAGAACC | GTTTGCGCTGCCGAATAACCAC |

**Supplementary Table 2** Prediction of potential *Drosophila* transcriptional factors binding to P3 DNA sequence by FIMO (Find Individual Motif Occurrences).

| Motif ID | Alt ID | Start | End | p-value | q-value | Matched Sequence |
| --- | --- | --- | --- | --- | --- | --- |
| FBgn0035454_2 | CG12029_SOLEXA_5 | 1176 | 1190 | 1.00E-06 | 0.00254 | TCTGGGTGTGGCTAT |
| FBgn0001994 | crp_SANGER_10 | 479 | 489 | 1.59E-06 | 0.00353 | AAACAGCTGAT |
| FBgn0035454 | CG12029_SANGER_10 | 1179 | 1189 | 1.68E-06 | 0.00438 | CTGGGTGTGGC |
| FBgn0034810_2 | CG9895_SOLEXA_5 | 1178 | 1188 | 1.95E-06 | 0.00505 | TGGGTGTGGCT |
| FBgn0001994 | crp_SANGER_10 | 491 | 501 | 2.71E-06 | 0.00353 | CAACAGCTGAT |
| FBgn0002985_3 | odd_NBT_2.5 | 1297 | 1305 | 2.72E-06 | 0.00695 | GCTACCGGA |
| FBgn0002985_2 | odd_NBT_1.5 | 1297 | 1305 | 2.72E-06 | 0.00709 | GCTACCGGA |
| FBgn0003448 | sna_FlyReg | 180 | 189 | 4.67E-06 | 0.012 | CCACTTGCTT |
| FBgn0038418 | pad_SANGER_5 | 1118 | 1126 | 6.03E-06 | 0.0157 | GGAGGGGTA |
| FBgn0013263 | Trl_FlyReg | 534 | 543 | 9.44E-06 | 0.0242 | TCGCTCTCTT |
| FBgn0263108 | BtbVII_SANGER_5 | 253 | 262 | 9.54E-06 | 0.0241 | CCTATGTATA |
| FBgn0037446 | CG10267_SANGER_5 | 217 | 226 | 1.05E-05 | 0.027 | AACAACACTG |
| FBgn0000286_5 | Cf2-PB_SOLEXA | 734 | 744 | 1.27E-05 | 0.0327 | ATATATGTAGA |
| FBgn0019650 | toy_FlyReg | 1020 | 1031 | 1.32E-05 | 0.0341 | CCGCTCATCCAC |
| FBgn0035160 | CG13897_SANGER_5 | 809 | 819 | 1.39E-05 | 0.0357 | TGAAGAGACAC |
| FBgn0034810 | CG9895_SANGER_10 | 1179 | 1190 | 1.87E-05 | 0.0487 | TCTGGGTGTGGC |
| FBgn0040765_2 | luna_SOLEXA_5 | 1178 | 1191 | 1.90E-05 | 0.0494 | TTCTGGGTGTGGCT |
| FBgn0011277 | HLH4C_da_SANGER_5 | 477 | 491 | 1.91E-05 | 0.0496 | CCAAACAGCTGATCC |
| FBgn0000413_11 | HLH4C_da_SANGER_5 | 477 | 491 | 1.91E-05 | 0.0496 | CCAAACAGCTGATCC |

The p-value of a motif occurrence is defined as the probability of a random sequence of the same length as the motif matching that position of the sequence with as good or better a score. The q-value of a motif occurrence is defined as the false discovery rate if the occurrence is accepted as significant.
